# AI information encounters and university students’ digital burnout: the mediating role of cognitive flexibility and the moderating effect of AI learning trust

**DOI:** 10.3389/fpsyg.2026.1897395

**Published:** 2026-09-07

**Authors:** Zongshuang Li, Mingxia Song

**Affiliations:** 1 Department of Marxism, Tonghua Normal University, Tonghua, China; 2 Department of Medicine and Health, Tonghua Normal University, Tonghua, China

**Keywords:** AI information encounter, AI learning trust, cognitive flexibility, digital burnout, university students

## Abstract

Generative artificial intelligence (AI) has become an integral part of educational environments, where university students often encounter algorithm-recommended information unexpectedly, known as AI information encounters. Such interactions frequently lead to increased cognitive load, and it is unclear whether the ongoing depletion of these cognitive resources is statistically linked to digital burnout among university students. This study utilizes the Stimulus–Organism–Response (SOR) framework as its analytical foundation, employing cognitive load theory to elucidate the mediating role of cognitive flexibility, and social cognitive theory to explain the moderating role of AI learning trust, thereby constructing a moderated mediation model. A cross-sectional questionnaire survey, based on 566 valid responses, is conducted and analyzed using the PROCESS macro program (Model 4, 15) to examine mediation and moderated mediation effects. The results reveal that: (1) AI information encounters are significantly positively correlated with digital burnout; (2) cognitive flexibility partially mediates this relationship, as AI information encounters negatively correlate with cognitive flexibility, while cognitive flexibility negatively correlates with digital burnout; (3) AI learning trust negatively moderates the direct path between AI information encounters and digital burnout, meaning that higher levels of AI learning trust weaken their positive association; (4) AI learning trust also negatively moderates the link between cognitive flexibility and digital burnout, indicating that higher AI learning trust diminishes the negative association between cognitive flexibility and digital burnout. This study highlights the psychological costs associated with involuntary technology use and suggests that educators in higher education should be cognizant of the cognitive resource consumption resulting from AI information encounters, and the interactive effects of AI learning trust on this relationship. Additionally, educators should encourage students to develop reasonable AI learning trust and acknowledge the risk of digital burnout linked to AI information encounters.

## 1 Introduction

Generative artificial intelligence (GenAI) has become deeply embedded in the learning life of contemporary university students. In this human–AI collaborative digital ecosystem, a new type of information exposure behavior—AI information encounters—is becoming increasingly common (Cam and Chung, 2025; Zhao et al., 2026). AI tools have been widely applied in academic search, course study, and knowledge expansion, significantly improving students’ information acquisition efficiency and fundamentally altering students’ information exposure methods and learning behavior patterns (Hirvonen et al., 2024). This encounter involves students encountering AI-generated information unexpectedly and unplanned during interactions with AI systems in educational activities. Manifestations include AI-prompted content recommendations, unforeseen discoveries in assisted searches, and additional information surpassing the initial questions during interactive sessions. In contrast to conventional active and intentional information retrieval, AI-powered learning environments facilitate encounters with information, making such occurrences exceedingly common (Chen and Xiao, 2025). Although AI information encounters can broaden learners’ knowledge boundaries and stimulate exploratory thinking, it also brings significant cognitive burdens. Meanwhile, digital burnout is increasingly prominent among university students, becoming a widely concerned psychological phenomenon in the digital learning era (Avcı, 2025). Digital burnout is defined as a contextual negative psychological state, encompassing the cognitive depletion, emotional exhaustion, and reduced learning efficacy experienced by college students who, in an environment characterized by frequent AI information encounters, continuously receive and process a substantial amount of diverse and redundant AI-generated content. Existing studies have confirmed that disordered and excessive digital information stimulation is the core antecedent variable leading to various digital learning psychological issues (Fu et al., 2020; Zhao et al., 2026). However, most studies focus on general online information exposure or users’ intentional use of digital tools and have yet to systematically explore whether AI algorithm-induced specific information encounter behaviors exacerbate the negative learning psychology of university students; the internal mechanism between the two still needs clarification.

Cognitive flexibility, a core ability that enables individuals to adjust cognitive strategies and adapt to complex informational environments, is crucial when managing large volumes of fragmented, cross-domain AI information (Yilmaz et al., 2025). The cognitive demands and resource depletion associated with digital technology use can adversely affect an individual’s cognitive flexibility. University students with limited cognitive flexibility are more susceptible to cognitive fatigue and burnout in digital learning environments (Avcı, 2025). Chen and Xiao (2025) have preliminarily confirmed that cognitive flexibility mediates the relationship between AI information encounters and creativity, providing empirical evidence for its role as a vital connecting mechanism between AI information encounters and learners’ psychological states. However, this study predominantly explores positive outcomes, such as creativity, and lacks empirical investigation into whether cognitive flexibility is also linked to negative psychological outcomes like digital burnout in information overload situations. Moreover, an individual’s level of trust in AI tools significantly influences their information processing strategies and interaction behaviors during the learning process, serving as a critical psychological boundary condition that determines the effects and experiences of AI use (Martín-Moncunill and Alonso Martínez, 2025; Zhang and Yu, 2025). Consequently, trust in AI tools may play a key moderating role in the “AI information encounter—cognitive flexibility—digital burnout” pathway, influencing whether passive information overload ultimately results in learning burnout.

In summary, while existing research has examined the relationship between exposure to digital information, cognitive ability, and learning burnout, and has explored trust in AI’s impact on learning behavior to some extent, it has not systematically elucidated the intrinsic mechanisms of AI information encounters, cognitive flexibility, and digital burnout. Nor has it tested the moderating effect of AI learning trust within these dynamics. Consequently, this study focuses on college students to develop a moderated mediation model aimed at clarifying the mediating path of “AI information encounters—cognitive flexibility—digital burnout” and the boundary role of AI learning trust. This effort seeks to provide a theoretical foundation and practical guidelines for optimizing human–AI collaborative learning experiences and mitigating digital burnout.

## 2 Literature review

### 2.1 AI information encounter

AI information encounters represent an extension of traditional information encounters within the AI context. These encounters refer to instances where users accidentally discover interesting, useful, or potentially valuable information during interactions with AI technologies, without actively seeking it (Chen and Xiao, 2025). In conventional network environments, random encounters are limited by “filter bubbles,” resulting in relatively homogeneous content. Conversely, AI information encounters are characterized by algorithmic guidance combined with an element of unexpected openness; AI systems suggest pertinent content based on user profiles. Still, cross-domain capabilities of large models often introduce unfamiliar information that exceeds expectations or varies from users’ original intent, leading to encounters that might be “algorithm-related but ineffective for the user” (Silvestri, 2024). The frequent occurrence of such ineffective serendipitous instances, compounded by the vast amounts of AI-generated information, can trap users in information overload and exacerbate information anxiety (Qiu et al., 2025). Presently, academic discussions predominantly highlight the positive effects of AI information encounters, such as fostering innovative thinking, knowledge expansion, and cognitive flexibility. However, the potential negative psychological impacts, particularly its intrinsic link to digital burnout, have not been systematically explored. Given AI information encounters’ prevalence as a high-frequency informational behavior in university students’ daily learning, it is crucial to thoroughly examine its impact on digital burnout to address the existing research gap.

### 2.2 Cognitive flexibility

Martin and Rubin (1995) define cognitive flexibility as the tendency of individuals to identify multiple options in a situation, maintain a willingness to adapt, and believe in their ability to adjust flexibly. Kienhues and Bromme (2011) noted that in information-rich environments, cognitive flexibility is expressed through an individual’s ability to interpret conflicting information adaptively. This capacity depends on beliefs about one’s abilities and understanding of the uncertainty inherent in scientific knowledge. When individuals lack this interpretive flexibility, they may experience persistent cognitive stress due to ineffective integration of conflicting information. In digital education, the protective role of cognitive flexibility has substantial empirical backing. Huang et al. (2026) identified cognitive flexibility as pivotal in buffering learning burnout from digital teaching, acting as a mediator between digital stress and burnout. Similarly, Sheikh et al. (2025) reported that individuals with low cognitive flexibility struggle to adapt to dynamically changing digital environments, leading to cognitive blockages, distraction, and emotional fatigue, thus worsening digital burnout. Alsaif et al. (2024) confirmed that individuals with high cognitive flexibility effectively adjust cognitive strategies and switch thinking modes, reducing the likelihood of cognitive rigidity in response to digital overload, information conflict, and frequent task switching, thereby alleviating academic stress and emotional exhaustion. Notably, the information environments in these studies primarily involve general digital platforms or active information retrieval, not AI-driven, unplanned information encounters. The unpredictability, frequency, and algorithm-driven nature of AI encounters may pose a more sustained challenge to cognitive flexibility. Theoretically, cognitive flexibility, considered a cognitive resource available during information processing (Borghesi et al., 2024), is susceptible to depletion from a sustained information load but remains recoverable and adaptable (Ionescu, 2012). This trait makes it an appropriate mediator between stressors and psychological outcomes. Thus, exploring its role as a mediator in the relationship between AI information encounters and digital burnout is theoretically significant.

### 2.3 Digital burnout

In the digital age dominated by artificial intelligence, digital burnout is defined as a state of psychological exhaustion characterized by cognitive fatigue, emotional depletion, and behavioral alienation, resulting from information overload, algorithmic pressure, and the imbalance in human–AI collaboration due to the prolonged use of AI and other digital technologies (Zhao et al., 2026). Empirical research by Feng et al. (2023) identified a significant positive correlation between the frequency of ChatGPT usage and levels of online learning burnout. Marsh et al. (2024) also observed that information overload in digital environments notably increases the level of individual emotional exhaustion, serving as a key factor in the emergence of digital burnout. Balaskas et al. (2025) further pinpointed that information overload and sustained cognitive demands caused by AI algorithmic personalization significantly induce digital burnout. Yang et al. (2024) introduced the concept of algorithmic fatigue, validating that exposure to homogenized information via algorithmic pushes can result in psychological exhaustion, thereby providing a theoretical foundation for the link between AI information encounters and digital burnout. Nonetheless, most of these studies focus on the perspective of active use or general information pressure, without systematically examining the mechanism through which algorithmic pushes influence digital burnout from the standpoint of unplanned information encounters. In essence, while existing evidence supports the notion that “excessive AI usage leads to burnout,” it struggles to address the specific question of “whether and how the passive reception of AI information encounters results in burnout.” This gap serves as the core focus of this study.

### 2.4 AI learning trust

AI trust is defined as the psychological and behavioral inclination of individuals to accept AI, trust its recommendations, share tasks and information, and depend on its decisions (Siau and Wang, 2018). However, existing AI trust frameworks often fail to account for the distinctive teaching and contextual factors of higher education (Lelescu et al., 2025). This study, therefore, defines university students’ “AI learning trust” as the rational cognition formed by learners about the capabilities, transparency, and limitations of AI tools in higher education scenarios, along with their willingness to accept its outputs and actively adjust their learning strategies. Students’ trust in AI constitutes a fundamental psychological factor influencing their adoption of technology and information use. Research on AI trust reveals multifaceted dimensions. On one hand, various studies confirm that trust can enhance technology adoption and information acceptance. Nazaretsky et al. (2025) found that university students’ trust in AI educational technology increases usage behavior through perceived usefulness. Bouebdallah and Youssef (2025) demonstrated that trust is a crucial determinant in the use of generative AI and the willingness to recommend it. In contexts of information-seeking, trust in AI-generated scientific information significantly impacts the evaluation and acceptance of such information (Greussing et al., 2025). On the other hand, some studies suggest that trust does not invariably yield positive outcomes. Panagopoulos et al. (2026) identified a paradox: despite the growing usage of generative AI among students, their trust in its outputs remains low, resulting in the phenomenon of “widespread use but lacking trust”. Ruan et al. (2026) and Sallam and Sallam (2025) found that trust can exacerbate technological stress through dependency paths, indicating that excessive trust might create negative effects. This divergence indicates that trust is not solely a facilitating factor. Theoretically, the moderating role of trust can be interpreted through the information adoption model (Sussman and Siegal, 2003), where trust levels shape the depth of individuals’ cognitive processing of information—high trust facilitates systematic integration, whereas low trust prompts defensive questioning. Consequently, AI learning trust may moderate the strength and direction of the relationship between AI information encounters and digital burnout. Existing research primarily examines the facilitative effects of trust on adoption behavior, with limited exploration of its moderating role in complex learning experiences, such as serendipitous information encounters and digital burnout, which requires empirical testing. Thus, this study introduces AI learning trust as a moderating variable, providing a theoretical foundation for subsequent analysis of its role in AI information encounters and digital burnout.

## 3 Theoretical foundation and hypothesis

### 3.1 Stimulus–Organism–Response framework

This study employs the Stimulus–Organism–Response (SOR) framework as the foundation for the theoretical model. Proposed by Mehrabian and Russell (1974), the framework posits that external environmental stimuli trigger individuals’ internal cognitive and emotional states, eventually leading to behavioral or psychological outcomes. In this context, “Stimulus” pertains to external environmental factors influencing individuals’ perceptions or cognition, “Organism” refers to internal cognitive, emotional, or motivational states that mediate the effects of stimuli on responses, and “Response” signifies the psychological or behavioral outcomes driven by these internal states. Recently, the SOR framework has been extensively applied in research concerning digital technology and student behavior. Tafesse et al. (2024) demonstrated that digital overload, an external stimulus, affects student engagement by inducing psychological states such as technostress and emotional exhaustion. Han et al. (2026) further leveraged the SOR framework to reveal that information overload leads to social media fatigue, which subsequently results in reflective smartphone disengagement. In this study, AI information encounters, representing persistent and unexpected informational interferences, are considered typical external stimuli; cognitive flexibility is identified as a renewable cognitive resource, altering in response to changes in internal cognitive states as per the SOR framework; digital burnout is regarded as the ultimate psychological response. The SOR framework offers a comprehensive structure for understanding how AI information encounters influence university students’ digital burnout. Furthermore, it accommodates both the indirect pathway through organisms (SOR) and the possibility of direct effects of stimuli on responses (SR) (Mu and Yi, 2024), facilitating a unified theoretical approach to assess both main and mediating effects.

### 3.2 Cognitive load theory

Cognitive Load Theory, first systematically proposed by Sweller (1988), asserts that human working memory has a very limited capacity. When information processing demands surpass this limit, cognitive overload occurs, impairing both learning and performance. Sweller’s pioneering study demonstrated that cognitive load during problem-solving significantly influences learning outcomes. When individuals process multiple information sources simultaneously, cognitive resources are dispersed, reducing learning efficiency. Chandler and Sweller (1991) further corroborated Cognitive Load Theory with the introduction of the “split-attention effect.” This effect occurs when learners must mentally integrate spatially or format-wise disparate information sources, generating additional cognitive load and impairing learning outcomes. In the context of AI interaction, this mechanism is particularly evident: AI information encounters, marked by unpredictability, fragmentation, and high frequency, present “split-attention information,” persistently occupying limited working memory and leading to cumulative cognitive overload.

The impact of cognitive overload on cognitive flexibility can be elucidated through the following logical sequence: Cognitive flexibility is a consumable and restorable cognitive resource (Franconeri et al., 2013), contingent on the limited capacity of working memory. Continuous information load depletes cognitive resources, leading to a decline in cognitive flexibility performance (Miyake et al., 2000). When AI-generated information persistently taxes working memory, the “cognitive bandwidth” necessary for monitoring thought processes, adjusting strategies, and shifting mental sets is constricted, hindering the individual’s ability to reorganize thoughts and adjust strategies flexibly. This reduces cognitive flexibility’s effectiveness and is associated with an individual’s adaptation capacity to information overload, thus contributing to digital burnout. Avcı (2025)‘s empirical research suggests that positive technology stress can indirectly mitigate academic burnout by enhancing cognitive flexibility, providing empirical support for the inverse relationship between cognitive flexibility and digital burnout. In summary, cognitive load theory offers a cognitive mechanism-level explanation for the “Stimulus (AI information encounter)—Organism (cognitive flexibility)—Response (digital burnout)” chain within the SOR framework: unexpected information surges exhaust the limited working memory capacity, inducing cognitive overload, which affects an individual’s ability to process information flexibly and is linked to the depletion of psychological resources. It is important to note that while cognitive load theory serves as a theoretical basis for explaining the relationship between AI information encounters and cognitive flexibility, cognitive load was not directly measured, and the related discussion remains a theoretical interpretation.

The unplanned and high-frequency nature of incidental information results in individuals being continuously exposed to unpredictable disruptions, which correlate positively with emotional exhaustion (Horvat and Tement, 2020). Research by Pang (2021) on digital platform users confirmed a positive correlation between intrusions of irrelevant information and information overload with cognitive fatigue among users. As emotional exhaustion and cognitive fatigue are key characteristics of digital burnout, these established relationships in digital platforms can reasonably be extrapolated to contexts involving AI-generated incidental information.

Building on the aforementioned theoretical framework, this study posits the following hypotheses:

H1: AI information encounters are positively correlated with digital burnout among university students.

H2: Cognitive flexibility partially mediates the relationship between AI information encounters and digital burnout. Specifically, there is a negative correlation between AI information encounters and cognitive flexibility, as well as between cognitive flexibility and digital burnout.

### 3.3 Social Cognitive Theory

Social Cognitive Theory, introduced by Bandura (1986), posits that human behavior is a dynamic system influenced by the interplay of personal factors (such as cognition and beliefs), behavior, and the environment, a concept known as triadic reciprocal determinism. Bandura (1986) emphasized that individuals do not merely respond passively to environmental stimuli; instead, they actively interpret, assign meaning, and select coping strategies through cognitive evaluation processes. In the context of Social Cognitive Theory, trust is considered an important cognitive disposition that affects how individuals assess the credibility of external information sources and manage their cognitive resources (Bandura, 2001). The theory further suggests that an individual’s belief system can mitigate the negative effects of environmental stressors. Consequently, this study regards AI learning trust as a moderating variable and applies Social Cognitive Theory to the study’s moderating hypothesis, leading to the derivation of the following logic.

When students possess high levels of AI learning trust, they are more likely to perceive AI information encounters as reliable learning resources rather than as potential cognitive interferences demanding caution. A systematic review by Aquilino et al. (2025) highlights that trust in AI significantly influences user interaction patterns with AI systems. Ding et al. (2026) introduced the concept of vigilant trust, illustrating that the dimension of trust differentiates individuals’ alertness and evaluation modes toward AI information. Individuals with high trust maintain a critically open mindset, whereas those with low trust tend to exhibit excessive vigilance. According to Social Cognitive Theory, an individual’s belief system can reshape their cognitive appraisal of environmental stimuli (Bandura, 2001). High trust acts as a “cognitive safety net” in AI information encounters, reducing the likelihood of triggering defensive alertness and uncertainty anxiety and thus enabling students to respond with a more composed mental state. Conversely, students with low trust may experience increased cognitive strain due to ongoing vigilance and verification behaviors (Lee et al., 2025). A cross-situational study by Luo et al. (2026) further confirmed that trust can moderate the positive association between information-related stress and psychological exhaustion. This analysis indicates that the levels of alertness and anxiety arousal are crucial situational factors determining whether information encounters strongly correlate with digital burnout. In high-trust contexts, where alertness is not easily triggered, information encounters are perceived as ordinary external stimuli, weakening the positive linkage with digital burnout. In contrast, in low-trust contexts, where alertness remains heightened, the positive association is reinforced. Therefore, the following hypothesis is proposed:

H3: AI learning trust negatively moderates the positive relationship between AI information encounters and digital burnout. Specifically, when AI learning trust is higher, the positive association between the two becomes weaker.

Social Cognitive Theory posits that the utilization of an individual’s abilities is contingent upon their belief system (Bandura, 2001). Research has demonstrated that cognitive flexibility serves as a crucial psychological resource in mitigating digital burnout, with a documented negative correlation between the two (An et al., 2025). Nonetheless, heightened AI learning trust systems can lead students to increasingly rely on structured outcomes provided by these systems, thereby transferring cognitive tasks to AI. This represents a specific form of cognitive dependence in digital learning environments (Gerlich, 2025). Consequently, it is plausible to speculate that this cognitive offloading may diminish the necessity for students to engage their cognitive flexibility actively, weakening its protective impact against digital burnout. In essence, elevated trust in AI could attenuate the negative association between cognitive flexibility and digital burnout. Therefore, the following hypothesis is proposed:

H4: AI learning trust negatively moderates the negative association between cognitive flexibility and digital burnout. Specifically, when AI learning trust is higher, this negative association becomes weaker.

H3 and H4 distinctly illustrate the moderating effects of AI learning trust on two separate targets: external information stimuli and internal psychological resources. These effects are not contradictory but rather demonstrate how the same trust mechanism functions differently in digital learning contexts. When encountering external information stimuli, trust acts as a buffer, mitigating the positive correlation between external interference and burnout (H3). Conversely, when utilizing internal cognitive flexibility, trust serves as a substitute, lessening individuals’ reliance on internal resources and consequently diminishing its protective effectiveness (H4). This dual pathway of ‘external buffering and internal substitution’ exemplifies how cognitive beliefs assign different regulatory weights to external and internal factors, as highlighted by Social Cognitive Theory.

## 4 Methods

### 4.1 Data collection

This study utilized a cross-sectional survey design to gather data from February to April 2026, focusing on university students from five universities in Jilin Province, China. This study employs convenience sampling due to the feasibility and homogeneity characteristics of the target population. Given the challenges in obtaining a complete sampling framework of university students from all colleges in Jilin Province, convenience sampling is a viable approach. The student group exhibits high homogeneity in age, cognitive development, and exposure to digital technology, which may mitigate the systematic bias introduced by non-random sampling and ensure basic representativeness of the sample for comparable groups.

Inclusion criteria for the sample were: full-time undergraduate enrollment at universities in Jilin Province; experience using generative AI tools during studies; and the capability to independently comprehend and complete all questionnaire items. Participants who did not meet these criteria were excluded. Furthermore, questionnaires lacking data on key variables, exhibiting patterned responses, or completed in excessively short times (less than 120 s) were removed from the analysis.

The questionnaire was administered and collected via the Questionnaire Star platform. An independent informed consent page was established before data collection, detailing the study’s purpose, content overview, response duration, and assurances of anonymity and confidentiality. Participation was voluntary, and withdrawal was allowed at any time without repercussions; consent was indicated by actively selecting an agreement option. The questionnaire included standardized instructions with clear completion requirements. The data collection process was entirely anonymous, with no identifiable information gathered.

The sample size was determined via relevant methodological literature. According to a simulation study by Sim and Kim (2026), a moderated mediation model requires a sample size of 200–230 to achieve a statistical power of over 80% at a moderate effect size. The study ultimately collected 566 valid responses, achieving an effective response rate of 87.08% as detailed in Supplementary Table 1, which is sufficient to satisfy the main statistical analysis requirements.

### 4.2 Measurements

All items in this study (see Supplementary Table 2) were adapted from established scales and contextually adjusted to fit the learning scenarios involving AI applications among college students. A 5-point Likert scale was employed (1 = completely disagree, 5 = completely agree). The measurement tools for each variable are as follows:

#### 4.2.1 AI information encounters

This study defines AI information encounters as the process in which individuals, while learning through GenAI, come across information unrelated to their current query objectives but which captures their attention, subsequently triggering a series of cognitive processing and behavioral response processes. This scale is adapted from Chen and Xiao (2025) and includes four dimensions: noticing, stopping, checking, and capturing. Example items are: “When using GenAI for learning, I notice content that is unrelated to my query goals”, “When engaging in interactive learning with GenAI, I will suspend the current query if I encounter information unrelated to the original learning objective”, “Even if the information presented by GenAI is unrelated to my initial learning query, I will still review it as long as it captures my attention”, “Even if it is not the learning content I initially queried from GenAI, I will share the interesting information generated by AI with classmates, friends, or teachers”.

#### 4.2.2 Cognitive flexibility

In this study, cognitive flexibility is defined as the ability of university students to comprehend information from multiple perspectives, adapt cognitive strategies dynamically, and effectively manage the learning process in AI-assisted educational contexts when confronted with a substantial amount of fluctuating information. This scale, adapted from Chen and Xiao (2025), comprises two dimensions: multi-perspectives and alternatives, and sense of control and adaptability. Example items are: “When facing a large amount of learning information pushed by AI, I can understand and evaluate it from multiple perspectives”, “When facing information overload caused by AI, I can control my learning pace and avoid being overwhelmed”.

#### 4.2.3 AI learning trust

This study defines AI learning trust as the propensity of university students to trust and adopt AI-generated learning information. This is demonstrated by their positive evaluation of the value of AI information and their willingness to continue using it despite potential uncertainties, such as occasional inaccuracies. The scale, adapted from Üstün et al. (2026), consists of four items. A sample item is: “I trust and adopt incidental AI learning information and suggestions to adjust my own learning decisions”.

#### 4.2.4 Digital burnout

This study defines digital burnout as the state of emotional exhaustion and cognitive fatigue experienced by university students in AI-assisted learning environments due to information overload. The scale, adapted from Zhao et al. (2026), encompasses two dimensions: emotional exhaustion and cognitive fatigue. Example items include: “Faced with the explosive influx of learning information from AI and the internet, I often feel irritable and lack motivation”, and “The vast amount of learning information presented by AI makes it difficult for me to filter and process, often leaving my mind in a state of confusion”.

### 4.3 Data analysis methods

This study employed SPSS 27.0, Amos 26.0, and the PROCESS macro (v4.3) for data analysis. Common method bias was assessed using Harman’s single-factor test and the Unmeasured Latent Method Construct (ULMC) approach. Subsequently, dual discriminant validity was evaluated using a combination of the Fornell–Larcker criteria and the HTMT ratio method. Descriptive statistics and Pearson correlation analyses were performed with |skewness| < 3 and |kurtosis| < 7 as reference criteria for normal distribution. Cronbach’s alpha coefficient was used to evaluate the internal consistency reliability of the scales, accepting *α* > 0.7 as adequate. Confirmatory factor analysis (CFA) tested the structural validity of the scales, with fit index standards detailed in Supplementary Table 3. Hypothesis testing was conducted using the PROCESS macro (Hayes, 2018). To control for potential confounding variables from demographic and technical backgrounds, grade, major, AI usage duration, and AI usage experience were included as covariates in all regression models. Simple mediation effects were tested using Model 4, and moderated mediation effects using Model 15, based on the significance of interaction terms and conditional indirect effects of the moderating variable at varying levels. All models utilized the bias-corrected Bootstrap method with 5,000 resamples; a 95% confidence interval not containing zero indicated a significant effect.

## 5 Results

### 5.1 Common method bias test

A Harman’s single-factor test was conducted to assess the potential presence of common method bias. The analysis revealed that the variance explained by the first factor was 28.919%, which is substantially below the critical threshold of 50%. Furthermore, the latent common method factor test results indicated that the changes in Tucker–Lewis Index (∆TLI = 0.008) and Comparative Fit Index (∆CFI = 0.009) were both significantly below the 0.1 increase threshold. Additionally, the changes in Root Mean Square Error of Approximation (∆RMSEA = 0.004) and Standardized Root Mean Square Residual (∆SRMR = 0.013) did not exceed the 0.05 decrease threshold. These findings suggest an absence of significant common method bias in the data of this study.

### 5.2 Composite reliability and convergent validity test

The composite reliability (CR) values for each latent variable range from 0.871 to 0.914, all surpassing the recommended threshold of 0.70, indicating excellent internal consistency of the scale. The average variance extracted (AVE) values for each latent variable range from 0.685 to 0.723, all exceeding the criterion of 0.50. This suggests that the observed variables effectively reflect their corresponding latent variables, thereby confirming good convergent validity (see Supplementary Table 4).

### 5.3 Discriminant validity test

A dual discriminant validity test, incorporating both the Fornell–Larcker criterion and the HTMT ratio method, was conducted (see Supplementary Tables 5, 6). The square roots of the average variance extracted (AVE) for each latent variable ranged from 0.828 to 0.850, which were all greater than the absolute values of their correlations with other variables (ranging from 0.156 to 0.521), satisfying Fornell and Larcker’s (1981) criteria. The HTMT values for all variables ranged from 0.223 to 0.754, remaining below the recommended threshold of 0.85 (Henseler et al., 2015). In summary, the discriminant validity of each latent variable is robust, with no significant overlap among them.

### 5.4 Reliability and validity analysis

This study employs Cronbach’s *α* coefficient to assess the internal consistency reliability of the scale (Table 1). The Cronbach’s *α* for each variable ranges from 0.836 to 0.902, demonstrating good internal consistency. The variance inflation factor (VIF) values for all variables range from 1.065 to 1.453, indicating no severe multicollinearity issues. The structural validity test reveals a KMO value of 0.897 and a significant Bartlett’s test of sphericity (*χ*^2^ = 11509.714, df = 561, *p* < 0.001). The results of the confirmatory factor analysis show that the overall measurement model meets acceptable fit standards: *χ*^2^/df = 2.211, GFI = 0.894, AGFI = 0.878, NFI = 0.904, TLI = 0.939, CFI = 0.945, RMSEA = 0.046, and SRMR = 0.048, with GFI and AGFI meeting only the acceptable threshold (≥0.85).

**Table 1:** Reliability test and variance inflation factor of each scale.

Variable	Cronbach’s *α*	VIF
AIIE	0.894	1.453
CF	0.902	1.438
DB	0.836	1.433
AILT	0.902	1.065

AIIE, AI information encounter; CF, cognitive flexibility; DB, digital burnout; AILT, AI learning trust.

### 5.5 Descriptive statistics and correlation analysis

The descriptive statistics, presented in Table 2, reveal that the mean scores for AI information encounter (M = 3.537, SD = 0.729), digital burnout (M = 3.490, SD = 1.221), and AI learning trust (M = 3.423, SD = 0.803) are at medium-high levels, whereas the mean score for cognitive flexibility (M = 2.299, SD = 0.799) is comparatively low. The absolute values of skewness and kurtosis conform to the criteria for normal distribution. The correlation analysis results, displayed in Table 3, indicate a significant negative correlation between AI information encounter and both cognitive flexibility (*r* = −0.486, *p* < 0.01) and AI learning trust (*r* = −0.177, *p* < 0.01), along with a significant positive correlation with digital burnout (*r* = 0.461, *p* < 0.01). Furthermore, cognitive flexibility is positively correlated with AI learning trust (*r* = 0.144, *p* < 0.01) and negatively correlated with digital burnout (*r* = −0.457, *p* < 0.01). Finally, AI learning trust shows a significant negative correlation with digital burnout (*r* = −0.233, *p* < 0.01).

**Table 2:** Descriptive statistics of core variables.

Variable	M	SD	Skewness	Kurtosis
AIIE	3.537	0.729	−0.732	0.212
CF	2.299	0.799	1.147	0.820
DB	3.490	1.221	−0.506	−1.160
AILT	3.423	0.803	−0.342	−0.608

M, mean; SD, standard deviation.

AIIE, AI information encounter; CF, cognitive flexibility; DB, digital burnout; AILT, AI learning trust.

**Table 3:** Correlations among variables.

Variable	AIIE	CF	AILT	DB
AIIE	1			
CF	−0.486^**^	1		
AILT	−0.177^**^	0.144^**^	1	
DB	0.461^**^	−0.457^**^	−0.233^**^	1

^*^Means *p* < 0.05, ^**^means *p* < 0.01, ^***^means *p* < 0.001.

AIIE, AI information encounter; CF, cognitive flexibility; DB, digital burnout; AILT, AI learning trust.

### 5.6 Main effect and mediation effect

The results from the path coefficient analysis (Table 4) demonstrate that, after controlling for related variables such as grade, major, AI usage duration, and AI usage experience, AI information encounters have a significant total effect on digital burnout (*β* = 0.482, *p* < 0.001). The model accounts for 23.9% of the total variance in digital burnout (*R*^2^ = 0.239), thus supporting H1. There exists a significant negative correlation between AI information encounters and cognitive flexibility (*β* = −0.510, *p* < 0.001), and cognitive flexibility is significantly negatively correlated with digital burnout (*β* = −0.287, *p* < 0.001). Upon introducing the mediating variable, the direct effect of AI information encounters on digital burnout remains significant (*β* = 0.335, *p* < 0.001). The comprehensive model explains 29.9% of the total variance in digital burnout (*R*^2^ = 0.299). In comparison to the model that includes only AI information encounters (*R*^2^ = 0.239), the inclusion of cognitive flexibility enhances the model’s explanatory power by 6.0% (Δ*R*^2^ = 0.060). The results of the Bootstrap mediating effect test (Table 5) indicate that the total effect [*β* = 0.481, 95% CI (0.401, 0.562)] and the direct effect [*β* = 0.335, 95% CI (0.247, 0.423)] of AI information encounters on digital burnout are both significant. Moreover, the indirect effect identified in this study is also significant [*β* = 0.146, 95% CI (0.094, 0.196)]. These findings suggest that cognitive flexibility partially mediates the relationship between AI information encounters and digital burnout, supporting H2.

**Table 4:** Path coefficients of the mediation model.

Path	*β*	*t*
AIIE → CF	−0.510^***^	−12.636
CF → DB	−0.287^***^	−6.926
AIIE → DB (total effect)	0.482^***^	11.695
AIIE → DB (direct effect)	0.335^***^	7.478

*β* = standardized regression coefficient, *t* = *t*-statistic. ***Means *p* < 0.001.

AIIE, AI information encounter; CF, cognitive flexibility; DB, digital burnout; AILT, AI learning trust.

**Table 5:** Bootstrap test results of mediating effects.

Effect type	*β*	95% CI
Total effect	0.481	[0.401, 0.562]
Direct effect	0.335	[0.247, 0.423]
Indirect effect	0.146	[0.094, 0.196]

*β* = standardized regression coefficient, 95% CI = 95% bootstrap confidence interval.

### 5.7 Moderated effect test

**Figure 1:**
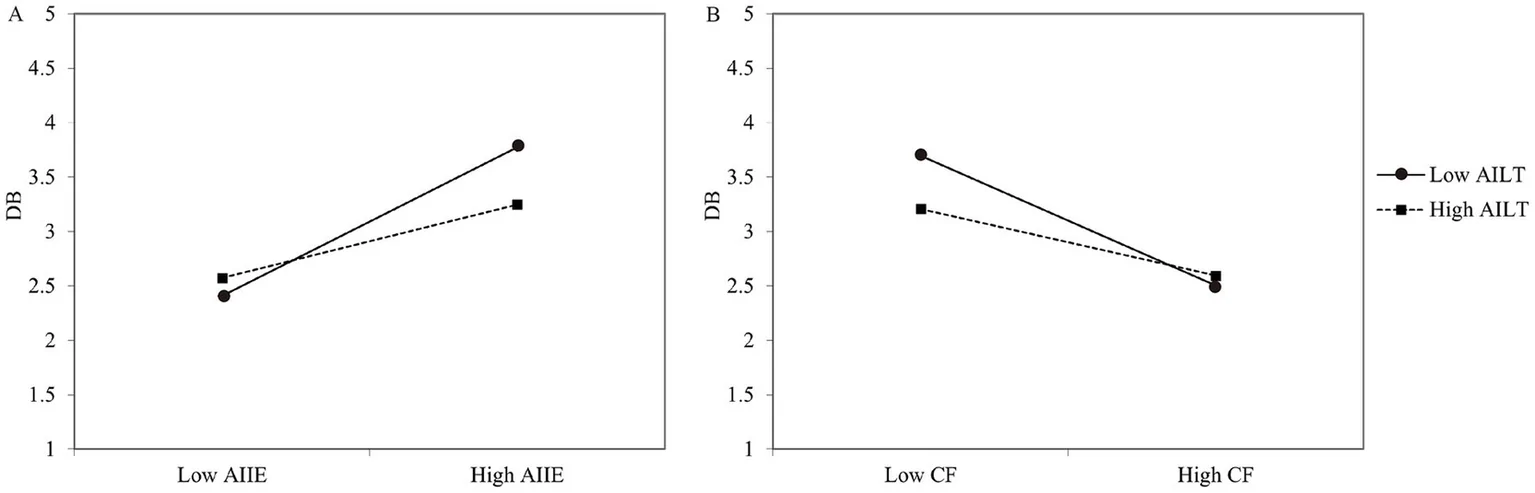
Simple slopes plot of AI learning trust as a moderator. **(A)** Simple slope analysis of the relationship between AI information encounters and digital burnout under the moderation of AI learning trust. **(B)** Simple slope analysis of the relationship between cognitive flexibility and digital burnout under the moderation of AI learning trust.

According to Table 6, the interaction term (AI information encounter × AI learning trust) has a significantly negative coefficient (*β* = −0.186, *t* = −4.994, *p* < 0.001). Combined with the results from Table 4, AI information encounters are significantly positively correlated with digital burnout. AI learning trust significantly moderates and negatively influences the positive relationship between AI information encounters and digital burnout. Including the interaction term in the model increases the explained variance by Δ*R*^2^ = 0.031, resulting in an overall model explanatory power of *R*^2^ = 0.294. The simple slope graph (Figure 1A) visually indicates that under conditions of low AI learning trust (solid line), digital burnout increases more sharply as AI information encounters rise from low to high levels, resulting in a steeper slope. Conversely, for the group with high AI learning trust (dotted line), the slope is gentler. This demonstrates that higher levels of AI learning trust significantly weaken the positive association between AI information encounters and digital burnout, thereby supporting hypothesis H3.

**Table 6:** Moderation effects of AI learning trust.

Moderated path	Interaction term	*β*	*t*	*R* ^2^	∆*R*^2^
AIIE → DB	AIIE × AILT	−0.186^***^	−4.994	0.294	0.031
CF → DB	CF × AILT	0.179^***^	4.842	0.287	0.029

*β* = standardized regression coefficient, *t* = *t*-statistic, *R*^2^ = explained variance, ∆*R*^2^ = change in *R*-squared.

AIIE, AI information encounter; CF, cognitive flexibility; DB, digital burnout; AILT, AI learning trust.

The interaction term (cognitive flexibility × AI learning trust) has a significantly positive coefficient (*β* = 0.179, *t* = 4.842, *p* < 0.001), and Table 4 shows that cognitive flexibility is significantly negatively correlated with digital burnout. Thus, the positive interaction coefficient suggests that AI learning trust positively moderates the relationship between cognitive flexibility and digital burnout. With the interaction term included, the model’s incremental variance is Δ*R*^2^ = 0.029, and the complete model *R*^2^ = 0.287. The simple slope graph (Figure 1B) shows both fitted lines trending downward, indicating that higher cognitive flexibility correlates with lower individual digital burnout. The slope for low AI learning trust (solid line) is steeper, signifying a stronger decrease in digital burnout with increased cognitive flexibility. Meanwhile, the slope for high AI learning trust (dotted line) tends to flatten. This suggests that as AI learning trust levels increase, the strength of the negative relationship between cognitive flexibility and digital burnout is diminished. Hence, hypothesis H4 is supported.

### 5.8 Moderated mediation effect test

Table 7 presents the moderation effects of varying levels of AI learning trust (low, medium, high) on the mediation pathway of “AI information encounter—cognitive flexibility—digital burnout”. The results demonstrate that at low, medium, and high levels of AI learning trust, the indirect effects of AI information encounters on digital burnout are significant: at a low level, the effect size is 0.214 with a 95% CI [0.145, 0.297]; at a medium level, the effect size is 0.128 with a 95% CI [0.077, 0.176]; and at a high level, the effect size is 0.099 with a 95% CI [0.025, 0.163]. Notably, all confidence intervals do not include zero. Furthermore, the moderated mediation effect index is −0.038 with a 95% CI [−0.082, −0.004], indicating a significant moderation by AI learning trust. Thus, as the level of AI learning trust increases, the indirect effect’s strength significantly diminishes.

**Table 7:** Moderated mediation effects of AI learning trust.

Path	AILT level	Effect	95%CI
AIIE → AILT → DB	AILT (low)	0.214	[0.145, 0.297]
	AILT (medium)	0.128	[0.077, 0.176]
	AILT (high)	0.099	[0.025, 0.163]
	Index of moderated mediation	−0.038	[−0.082, −0.004]

AIIE, AI information encounter; CF, cognitive flexibility; DB, digital burnout; AILT, AI learning trust.

**Figure 2:**
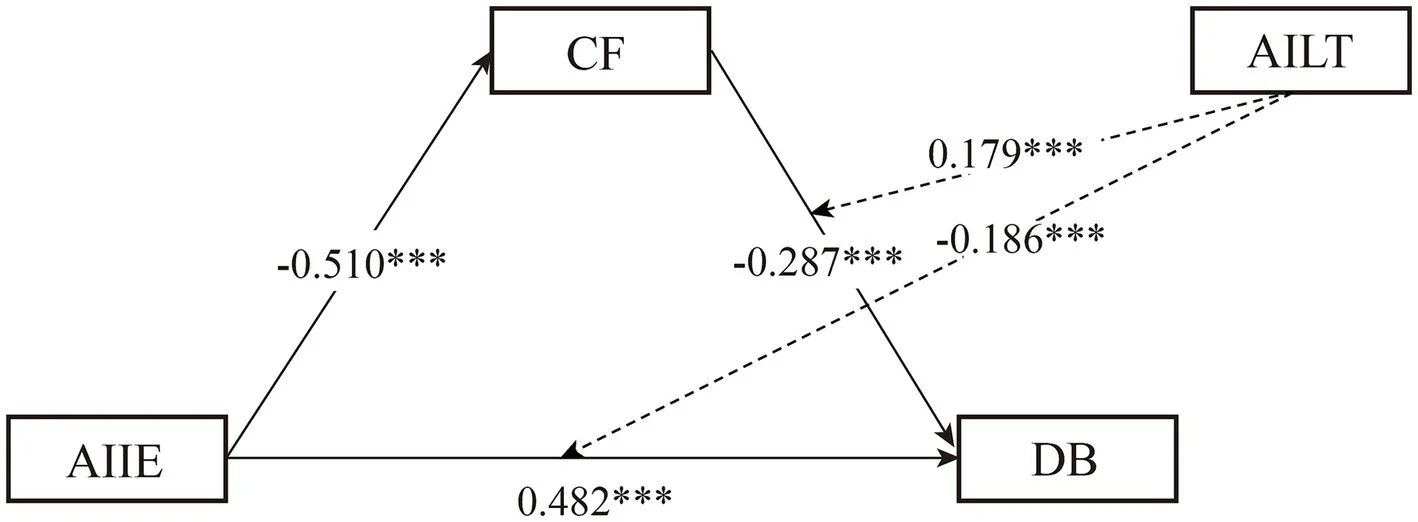
The moderated mediation model of AI information encounters on digital burnout.

Figure 2 illustrates that the overall model is validated by the data. There is a significant positive correlation between AI information encounters and digital burnout. The indirect effects reveal a negative correlation between AI information encounters and cognitive flexibility, as well as between cognitive flexibility and digital burnout. The latter segment of this mediation path is notably moderated by AI learning trust. Higher levels of AI learning trust result in a weaker negative correlation between cognitive flexibility and digital burnout, consequently reducing the mediating effect’s strength. Additionally, AI learning trust significantly negatively moderates the positive correlation between AI information encounters and digital burnout.

## 6 Discussion

### 6.1 Analysis of the main effect of AI information encounters on digital burnout

The study identified a significant positive correlation between AI information encounters and digital burnout among university students (H1), with the empirical results aligning with the hypothesized direction. This finding logically conforms to the theoretical expectations of the SOR framework, indicating that external environmental stimuli can be directly linked to individual behaviors or psychological responses (Mu and Yi, 2024). In this context, AI information, due to its unexpected and high-frequency characteristics, serves as an external stimulus distinct from proactive AI usage. From a cognitive standpoint, cognitive load theory further elucidates this direct correlation: unexpected, fragmented information often brings a persistent cognitive burden, with each encounter depleting cognitive resources. This effect mirrors the “split-source information effect” proposed by Chandler and Sweller (1991). Makri and Buckley (2020) identified the procedural source of this cognitive burden, requiring users to conduct a “highly uncertain cost-benefit analysis” when encountering unexpected information. This uncertainty-driven decision-making adds to the cognitive load, potentially accelerating mental resource depletion and triggering fatigue during frequent encounters. Recent studies validate this association pattern in various contexts. Guo et al. (2024) empirically established that frequent social media push notifications, as passively invasive external information, significantly correlate with user fatigue. Similarly, Nguyen and Nguyen (2026) found a significant positive correlation between information overload in AI content push environments and social media fatigue. Although focusing on different scenarios, these studies converge on a common conclusion: a significant covariant trend exists between unexpected information exposure and psychological exhaustion, providing a robust cross-contextual reference for the direct correlation anticipated by H1. In summary, this study not only verifies the applicability of the SOR framework and cognitive load theory in the setting of AI information encounters but also integrates “AI information encounters” as an external stimulus into the digital burnout correlation model. This finding suggests that the SOR framework has explanatory power in contexts of non-active technology use. It also encourages extending the research focus on AI-related technology stress from the premise of “how users engage with AI” to scenarios of “AI information encounters.” The latter is noteworthy for its potential link to psychological costs.

### 6.2 Analysis of the mediating effect of cognitive flexibility

This study identifies cognitive flexibility as a partial mediator between AI information encounters and digital burnout in university students, elucidating a key cognitive pathway from external stimuli to adverse reactions. AI information encounters consistently consume individuals’ working memory resources, diminishing their capacity to monitor thought processes, adjust strategies, and switch cognitive frameworks. This depletion of cognitive functions exacerbates digital burnout. The findings support the SOR framework’s emphasis on the mediating function of the “organism” and offer a procedural explanation of the psychological costs associated with AI information encounters. Wu et al. (2026) theorize that cognitive flexibility is a pivotal resource for learners navigating complex, multi-source information in AI environments. Individuals with elevated cognitive flexibility can adaptively adjust their information processing strategies, thereby reducing cognitive load and negative learning experiences. Further, Kaya and Keskin (2026) demonstrate that cognitive flexibility serves as a critical protective factor against negative psychological experiences in AI contexts: higher levels of cognitive flexibility correlate with increased self-efficacy and acceptance of AI, alongside decreased AI-related anxiety. This “high cognitive flexibility-low anxiety” relationship provides crucial empirical support for the mediating role of cognitive flexibility in the relationship between AI information encounters and digital burnout. Previous research indicates that serendipitous encounters with AI information can positively predict creativity under specific conditions, with cognitive flexibility serving as a positive mediator (Chen and Xiao, 2025). While this appears to contradict the “depletion effect” observed in this study, it actually suggests that the mediating role of cognitive flexibility may depend on information characteristics and individual processing modes. Structured, task-related encounters may enhance cognitive capabilities, whereas the unexpected and fragmented nature of AI information encounters in this study is more likely to impose an external cognitive load, depleting rather than enhancing cognitive flexibility. Zhang et al. (2026) provide further support with their cognitive mediation model, showing that the effect of incidental AI information exposure on cognitive outcomes depends on the depth of an individual’s elaborative processing. Deep processing facilitates cognitive growth, whereas shallow processing may increase cognitive burdens. These findings suggest that the mediating role of cognitive flexibility may be moderated by an individual’s cognitive processing mode, warranting further investigation into this boundary condition. In summary, this study contributes by extending the examination of cognitive flexibility’s mediating effect to the context of “unexpected, fragmented” AI information encounters, elucidating the mechanism between the AI information environment and digital burnout, and enriching the understanding of the “organism” component within the SOR framework. Despite controlling for numerous variables, this study cannot entirely rule out the influence of omitted variables, such as personality traits and academic stress. These unmeasured factors may simultaneously impact both AI information encounter and digital burnout. Future research should consider including these variables to strengthen the robustness of the findings.

### 6.3 Analysis of the moderating effect of AI learning trust

The study found that AI learning trust negatively moderates the positive relationship between AI information encounters and digital burnout (H3). This indicates that AI learning trust, as a vital psychological resource, can significantly act as a buffer between AI information encounters and digital burnout. This finding aligns with social cognitive theory (Bandura, 1986), which posits that individuals’ beliefs about technology can regulate the impact of external environmental stimuli on psychological and behavioral outcomes. It also reflects the established consensus in technology usage research: an individual’s level of trust in technology significantly influences their coping strategies and psychological responses to technology-related external stimuli (Ruan et al., 2026). Zhou et al. (2024) found that employees with higher trust in AI are more likely to adopt proactive learning strategies instead of passive avoidance when facing AI-induced work challenges, thereby mitigating the negative effects of technological environments. Similarly, this study observed that students with high AI learning trust experience less information overload anxiety; they do not need to engage in high-intensity scrutiny of AI-generated content, thus reducing unnecessary cognitive load. From the perspective of cognitive load theory, trust functions as an efficient “cognitive energy-saving mechanism” (Peng and Yeh, 2025). When individuals trust AI’s ability to filter and recommend information, they can conserve cognitive resources for core learning rather than exhaust them on in-depth processing and verification, thus avoiding burnout from excessive cognitive resource consumption. Lee et al. (2025) further corroborated this by showing that continuously questioning AI-generated content consumes substantial cognitive resources, which, when depleted, reduces criticality and leads to a “doubt-exhaustion-submission” cycle. This suggests that excessive skepticism and scrutiny can deplete cognitive resources and exacerbate digital burnout. Learners with higher trust levels can avoid the cognitive burden associated with “over-vigilance,” maintaining lower levels of cognitive load and burnout despite the same degree of information exposure. Therefore, AI learning trust is a crucial psychological resource that buffers the effects of AI information encounters on digital burnout.

Building on this foundation, this research not only examines the direct effects of AI learning trust regulation but also uncovers the negative moderating role of AI learning trust on the relationship between cognitive flexibility and digital burnout (H4). This finding is consistent with the interaction logic of individual beliefs and cognitive traits espoused by social cognitive theory, thereby extending its application to intelligent learning contexts. According to Bandura’s (2001) triadic reciprocal determinism, there is a dynamic interplay among personal factors (cognitive flexibility), environmental factors (AI learning trust), and behavioral outcomes (digital burnout). The study reveals that AI learning trust, as a situational moderating variable, reshapes the pathways between personal factors and behavioral outcomes. To elucidate the internal mechanism of this interaction, the study introduces the theory of cognitive offloading as a supplementary perspective, providing a possible cognitive mechanism interpretation for H4. When learners place a high level of trust in AI, the technological environment shifts from a “passive tool” to a “cognitive agent,” prompting individuals to outsource cognitive processes such as information filtering, judgment formation, and strategy adjustment to the system, resulting in cognitive outsourcing behavior. Risko and Gilbert (2016) noted that individuals use external tools to store or process information, thereby conserving internal cognitive resources. However, excessive reliance on external cognitive tools like AI systems may lead to cognitive laziness, reducing individuals’ propensity for reflective thinking and analytical reasoning (Yang et al., 2025). In this state, individuals with high AI learning trust no longer utilize their flexible cognitive strategies to manage large volumes of information, diminishing the protective role of cognitive flexibility. In summary, environmental factors (AI learning trust) modify the conditions and necessity for personal factors (cognitive flexibility) to function, relegating it from a “necessary capacity” to a “backup resource,” thereby negatively moderating its negative association with digital burnout. This finding highlights the potential complex functional substitution relationship between individual cognitive resources and technological trust in intelligent learning contexts.

The combined empirical results of H3 and H4 reveal that AI learning trust follows a dual regulatory pathway characterized by “external buffering and internal substitution” within digital learning environments. This outcome aligns with social cognitive theory (Bandura, 2001), which predicts that cognitive beliefs hold different regulatory weights on internal and external factors. High levels of AI learning trust assist learners by preventing the cognitive drain associated with excessive vigilance, yet this trust may also foster over-reliance on AI systems. This reliance decreases the activation of cognitive strategies, thereby diminishing the protective role of cognitive flexibility. These effects are not contradictory but represent differentiated expressions of the same trust mechanism targeting different functional objectives: the former influences the intensity of external information stimuli, whereas the latter affects the need to engage internal cognitive resources. This finding proposes a “moderate range” of trust, suggesting that while it conserves cognitive energy, it might also pose the risk of fostering cognitive inertia. Future research could explore this threshold effect further using nonlinear tests or longitudinal designs.

The moderated mediation effect size reported in this study [effect = −0.038, 95% CI (−0.082, −0.004)] does not include zero within its confidence interval, thus it is statistically significant despite its small magnitude. Practically, this limited effect size indicates that AI learning trust is not a decisive factor in reducing digital burnout. Enhancing AI learning trust can only partially mitigate the positive correlation between AI information encounters and digital burnout, without altering the core mediating role of cognitive flexibility within this process. These findings suggest that educational interventions should not solely emphasize cultivating learners’ AI learning trust but also concurrently focus on developing learners’ deep information processing and critical thinking skills to alleviate the continuous drain of cognitive resources by AI information encounters. Importantly, even small effect sizes can have significant practical impacts over time (Götz et al., 2022). The main value of this study is not in establishing a “strong moderation” effect of trust but in unveiling its differentiated moderation mechanisms at various stages within the SOR framework. Specifically, these findings illuminate the dual pathways of “external buffering” and “internal substitution,” offering a new theoretical perspective for understanding the dual role of trust in intelligent learning environments. This deepens the theoretical insights of social cognitive theory regarding the differentiated moderation roles of cognitive beliefs on external and internal factors.

### 6.4 Theoretical contributions

This study offers the following theoretical contributions: (1) While existing research often emphasizes the cognitive benefits of AI information encounter, this study reveals a significant positive correlation between AI information encounter and digital burnout among university students, thus providing empirical evidence for a dialectical analysis in information behavior research and enhancing the overall framework of information encounter. (2) Through the introduction of cognitive flexibility as a mediating variable, the study identifies a key explanatory pathway between AI information encounter and digital burnout. This expands the theoretical understanding of how intelligent information environments can implicitly erode individual cognitive abilities, moving beyond a sole focus on information overload and task load as explanations for digital burnout. (3) The study discovers that AI learning trust exerts a dual moderating effect, elucidating the varied relationships among variables through the lens of psychological trust. This widens the applicability of human-AI interaction trust theories and provides new evidence for exploring trust protection mechanisms in the digital health field. (4) By integrating cognitive traits and psychological trust elements into a moderated mediation model, the study systematically identifies the associative pathways and boundary conditions of digital burnout, offering theoretical and empirical support for future research in the field of intelligent educational psychology.

## 7 Conclusion

This study employs the SOR framework, integrating cognitive load theory and social cognitive theory, to investigate the relationship between AI information encounters and digital burnout among university students, as well as the mechanisms underpinning this relationship. The findings reveal a significant positive association between AI information encounters and digital burnout, with cognitive flexibility acting as a partial mediator. Additionally, AI learning trust exhibits a dual moderating effect in the latter stages of both the direct and mediating pathways: it mitigates the positive relationship between AI information encounters and digital burnout, while also reducing the negative connection between cognitive flexibility and digital burnout. These results extend the applicability of the SOR framework to educational technology, providing fresh insights into psychological experiences in non-proactive technology usage scenarios, and offering both theoretical foundations and practical implications for preventing and addressing digital burnout among university students.

This study has several limitations. First, the research employs a cross-sectional survey design that can only demonstrate correlations between variables, thus precluding any determination of causal relationships and failing to capture the dynamic changes of these variables. Second, the sample is limited to five universities in Jilin Province and was selected using convenience sampling, which restricts the generalizability of the findings. Third, all data were obtained through self-reported measures, particularly involving scales on cognitive flexibility and digital burnout. Participants’ perceptions of their cognitive performance may not accurately represent their actual cognitive levels. Moreover, self-reports are prone to recall bias, social desirability bias, and subjective interpretation errors, potentially leading to an overestimation or underestimation of the strength of associations between variables. Although common method bias was assessed, and findings indicated that it did not reach significant levels to compromise the reliability of core conclusions, it is not possible to entirely eliminate the potential impact of these biases due to the limitations inherent in the data collection method.

To address the aforementioned limitations, future research can be expanded and optimized in several ways: (1) In research design, employing a longitudinal design or cross-lag analysis can clarify the temporal relationships among variables through multi-time point data collection. Furthermore, integrating laboratory experiments can test causal relationships between variables while controlling for confounding variables, thereby revealing the dynamic patterns of how AI-related information impacts university students’ digital burnout. (2) Regarding sampling, the scope can be broadened to include university students from diverse regions and institutional levels, utilizing stratified or multi-stage sampling to enhance sample representativeness. This approach will increase the external validity and cross-context applicability of research findings. (3) For data collection, future studies can incorporate objective cognitive assessments (e.g., cognitive task performance, reaction time) or behavioral observation indicators (e.g., AI platform usage patterns) to corroborate self-reported findings. In addition, using multi-source data (e.g., teacher or peer evaluations) or physiological indicators (e.g., heart rate variability) for cross-verification can further minimize response bias and enhance the objectivity and reliability of the data.

## Data Availability

The raw data supporting the conclusions of this article will be made available by the authors, without undue reservation.
