# Supplementary material for: AI information encounters and university students’ digital burnout: the mediating role of cognitive flexibility and the moderating effect of AI learning trust
Source: Front Psychol. 2026 Sep 7;17:1897395. doi: 10.3389/fpsyg.2026.1897395 (PMC13590776; doi:10.3389/fpsyg.2026.1897395)
Supplement: Supplementary file 1 [file Supplementary_file_1.DOCX]

Supplementary Material

# Supplementary Table1 Demographic Characteristics of Participants

| Variable | Category | Frequency | Percentage |
| --- | --- | --- | --- |
| Grade | Freshman | 150 | 26.50% |
|  | Sophomore | 185 | 32.69% |
|  | Junior | 125 | 22.08% |
|  | Senior | 106 | 18.73% |
| Major | Humanities and Social Sciences | 261 | 46.11% |
|  | Science and Engineering | 305 | 53.89% |
| AI Usage Duration | 30 minutes–1 hour | 105 | 18.55% |
| (Daily usage time) | 1–2 hours | 169 | 29.86% |
|  | 2–3 hours | 231 | 40.81% |
|  | Over 3 hours | 61 | 10.78% |
| AI Usage Experience | 3–6 months | 86 | 15.19% |
| (Cumulative usage duration) | 6–12 months | 144 | 25.44% |
|  | 1–2 years | 336 | 59.36% |
| Total |  | 566 | 100% |

# Supplementary Table 2 Scale

**AI information encounter**

**Dimension 1** **noticing**

1. When using GenAI for learning, I notice content that is unrelated to my query goals.

2. During interactive learning with GenAI, I encounter information that I did not actively seek.

3. When using GenAI for learning, I find additional information generated by AI that I did not actively request.

4. During interactive learning with GenAI, I come across unexpected content beyond the scope of my initial learning needs.

**Dimension 2** **stopping**

5. When engaging in interactive learning with GenAI, I will suspend the current query if I encounter information unrelated to the original learning objective.

6. During the process of interactive learning with GenAI, I will pause to review information unrelated to the original learning query.

**Dimension 3** **checking**

7. Even if the information presented by GenAI is unrelated to my initial learning query, I will still review it as long as it captures my attention.

8. When engaging in interactive learning with GenAI, if I come across interesting or unexpected information, I will ask the AI for more related content.

9. When engaging in interactive learning with GenAI, if I come across interesting or unexpected information, I will open a new tab or window to further explore it.

**Dimension 4** **capturing**

10. Even if it is not the learning content I initially queried from GenAI, I will share the interesting information generated by AI with classmates, friends, or teachers.

11. When I come across interesting content during interactions with GenAI, I will apply it to ongoing learning tasks or course projects.

12. I will inform classmates and teachers about the interesting information generated by GenAI that is helpful for learning.

**Cognitive flexibility**

**Dimension 1** **multi-perspectives and alternatives**

1. When facing a large amount of learning information pushed by AI, I can understand and evaluate it from multiple perspectives.

2. When encountering different viewpoints provided by AI, I am willing to consider various interpretations and possibilities.

3. In regard to the complex learning content provided by AI, I can flexibly switch my ways of thinking.

4. When the information given by AI is contradictory, I can find reasonable ways to integrate it.

5. I can flexibly employ AI to acquire different types of information depending on the learning context.

6. When dealing with AI-related learning tasks, I can come up with diverse approaches to solve problems.

**Dimension 2:** **sense of control and adaptability**

7. When facing information overload caused by AI, I can control my learning pace and avoid being overwhelmed.

8. When a large amount of learning information from AI floods in, I do not feel confused or incapable of coping.

9. I am confident in my ability to effectively manage the information load brought by AI.

10. I can adapt to a learning environment characterized by the constant information stream from AI.

11. Even with a high volume of AI-generated learning information, I can maintain clear thinking and judgment.

**AI learning trust**

1. I trust and adopt incidental AI learning information and suggestions to adjust my own learning decisions.

2. I believe that incidental AI learning information and suggestions can help improve the quality of my learning.

3. I am willing to use incidental AI learning information and suggestions as a supplementary reference in my learning process.

4. Even if I occasionally find slight inaccuracies in incidental AI learning information and suggestions, I am still willing to continue referring to such information.

**Digital burnout**

**Dimension 1:** **emotional exhaustion**

1. Faced with the explosive influx of learning information from AI and the internet, I often feel irritable and lack motivation.

2. Digital learning tasks relying on AI make me feel constant pressure, even to the extent of developing aversion to studying.

3. After prolonged online learning with the help of AI, I often feel downhearted and lose interest in various activities.

**Dimension 2:** **cognitive** **fatigue**

4. The vast amount of learning information presented by AI makes it difficult for me to filter and process, often leaving my mind in a state of confusion.

5. The excessive input of AI-related learning information hinders my ability to think deeply, and I also experience a decline in memory.

6. Handling multiple AI-based online learning tasks simultaneously makes it hard for me to concentrate.

7. Frequent exposure to AI learning information and usage of digital devices severely affects my ability to focus on studies, leading to task procrastination.

# Supplementary Table 3 Evaluation Criteria of Confirmatory Factor Analysis Fit Indices

| Fit Index | Good fit criteria | Acceptable fit criteria |
| --- | --- | --- |
| χ²/df | 0 ≤ χ²/df ≤ 3 | 3 ≤ χ²/df ≤ 5 |
| GFI | 0.90 ≤ GFI | 0.85 ≤ GFI |
| AGFI | 0.90 ≤ AGFI | 0.85 ≤ AGFI |
| NFI | 0.90 ≤ NFI | 0.80 ≤ NFI |
| TLI | 0.90 ≤ TLI | 0.80 ≤ TLI |
| CFI | 0.90 ≤ CFI | 0.85 ≤ CFI |
| RMSEA | 0.00 ≤ RMSEA ≤ 0.05 | 0.05 ≤ RMSEA ≤ 0.08 |
| SRMR | 0.00 ≤ SRMR ≤ 0.05 | 0.05 ≤ SRMR ≤ 0.08 |

Note: *χ*^2^= Chi-squared, *df* = Degrees of Freedom, *GFI* = Goodness of Fit Index, *AGFI* = Adjusted Goodness of Fit Index, *NFI* = Normed Fit Index, *TLI* = Non-Normed Fit Index, *CFI* = Comparative Fit Index, *RMSEA* = Root Mean Square Error of Approximation, *SRMR* = Standardized Root Mean Square Residual

**Supplementary Table 4 Composite Reliability (CR) and Average‑Variance‑Extracted (AVE)**

| Latent variable | CR | AVE |
| --- | --- | --- |
| AIIE | 0.914 | 0.723 |
| CF | 0.887 | 0.698 |
| DB | 0.871 | 0.685 |
| AILT | 0.905 | 0.702 |

**Supplementary Table 5 Fornell‑Larcker discriminant‑validity matrix**

| Latent construct | AIIE | CF | AILT | DB |
| --- | --- | --- | --- | --- |
| AIIE | 0.850 |  |  |  |
| CF | -0.497 | 0.835 |  |  |
| AILT | -0.198 | 0.156 | 0.838 |  |
| DB | 0.483 | -0.521 | -0.318 | 0.828 |

**Supplementary Table 6 HTMT results for discriminant validity**

| Discriminant validity analysis | AIIE | CF | AILT | DB |
| --- | --- | --- | --- | --- |
| AIIE | —— |  |  |  |
| CF | 0.700 | —— |  |  |
| AILT | 0.278 | 0.223 | —— |  |
| DB | 0.686 | 0.754 | 0.458 | —— |
